# Catabolic Machinery of the Human Gut Microbes Bestow Resilience Against Vanillin Antimicrobial Nature

**DOI:** 10.3389/fmicb.2020.588545

**Published:** 2020-10-16

**Authors:** Monika Yadav, Rajesh Pandey, Nar Singh Chauhan

**Affiliations:** ^1^Department of Biochemistry, Maharshi Dayanand University, Rohtak, India; ^2^Genomics and Molecular Medicine, CSIR-Institute of Genomics and Integrative Biology (CSIR-IGIB), New Delhi, India

**Keywords:** human gut microbes, food metabolism, vanillin catabolism, food additives, metabolomics, metagenomics, antimicrobial resistance

## Abstract

Vanillin is a phenolic food additive commonly used for flavor, antimicrobial, and antioxidant properties. Though it is one of the widely used food additives, strategies of the human gut microbes to evade its antimicrobial activity await extensive elucidation. The current study explores the human gut microbiome with a multi-omics approach to elucidate its composition and metabolic machinery to counter vanillin bioactivity. A combination of SSU rRNA gene diversity, metagenomic RNA features diversity, phylogenetic affiliation of metagenome encoded proteins, uniformly (*R* = 0.99) indicates the abundance of Bacteroidetes followed by Firmicutes and Proteobacteria. Manual curation of metagenomic dataset identified gene clusters specific for the vanillin metabolism (*ligV, ligK*, and *vanK*) and intermediary metabolic pathways (*pca* and *cat* operon). Metagenomic dataset comparison identified the omnipresence of vanillin catabolic features across diverse populations. The metabolomic analysis brings forth the functionality of the vanillin catabolic pathway through the Protocatechuate branch of the beta-ketoadipate pathway. These results highlight the human gut microbial features and metabolic bioprocess involved in vanillin catabolism to overcome its antimicrobial activity. The current study advances our understanding of the human gut microbiome adaption toward changing dietary habits.

## Introduction

The human gut microbiome is a stratified, metabolically active, and resilient biotic component of the human body (Yadav et al., 2018). Its progressive establishment starts with human birth and matures by the adulthood of the host (Vemuri et al., 2018). The gut microbes continuously interact, mostly as commensals with the host for their survival (Parker et al., 2018) and maintenance of the healthy host physiology (Yadav et al., 2018). An adult gut microbiome is primarily enriched with Firmicutes and Bacteroidetes (Chauhan et al., 2018). The human gut microbiome enriches the host gene pool with additional 300,000 + genetic features to enrich the metabolic potential of the host (Qin et al., 2010; Oliphant and Allen-Vercoe, 2019). Role of the gut microbes in food digestion is being elucidated for decades, however, their role in xenobiotic/drug metabolism has only been recently discovered (Clarke et al., 2019). Human gut microbes are equipped with efficient metabolic machinery to metabolize polyphenols (Theophylline and Caffeine) (Yu et al., 2009) as well as pharmaceutical drugs (Zimmermann et al., 2019). Despite these discoveries, research toward unveiling the role of human gut microbes in the metabolism of food additives needs further attention.

Vanillin is the principal flavor and aroma component of the vanilla beans (Ramachandra Rao and Ravishankar, 2000). In addition to the flavoring properties, is also characterized by antimicrobial, antifungal, antioxidant, anticlastogenic, and antitumor properties (Durant and Karran, 2003; Shyamala et al., 2007; Sinha et al., 2008; Guo et al., 2018). Due to its flavoring and medicinal properties, it was used in Indian food preparations for many centuries (Menon and Nayeem, 2013). The natural preservative and flavoring properties of vanillin make it a commonly used food additive (Fitzgerald et al., 2004). Additionally, the degradation of lignin compounds also contributes (Furuya et al., 2015; Chen et al., 2016) to the availability of vanillin (10 mg/kg of the body weight in the human body) (Furuya et al., 2015; Chen et al., 2016). Continuous exposure of the vanillin exposes human gut microbes toward its antimicrobial property (Fitzgerald et al., 2004; Endo et al., 2009). It possibly challenges human gut microbes to either develop efficient metabolic machinery to respond to antimicrobial behavior of vanillin or a compromised survival trajectory. If microbes were unable to efficiently metabolize vanillin, it could lead to microbial dysbiosis, followed by the onset of microbial dysbiosis associated human disorders. On the contrary, vanillin is known to improve the gut microbiome composition (Guo et al., 2018), as well as protect the host from the onset of various human disorders (Yan et al., 2017). Thus, it indicates the possible presence of efficient vanillin catabolic machinery among the human gut microbes; however, an effort to explore these metabolic pathways is warranted. In the current study, we have explored the human gut microbiome composition, its genetic content, and metabolic efficiency to catabolize vanillin using a culture-independent multi-omics approach. This is a pioneer study to rationalize the role of human gut microbes in vanillin catabolism. The findings of this study hold potential to enrich our understanding of the gut microbial functionaries in the xenobiotic metabolism and evolution of the human gut microbiome with dietary habits.

## Materials and Methods

### Ethics Statement

The study was conducted after receiving ethical clearance from the Human Ethical Committee at M. D. University, Rohtak, Haryana, India. Strict human ethical guidelines were followed and written consent was sought from each enrolled individual in this study.

### Metagenomic DNA Isolation

The fecal samples were collected from healthy individuals (*n* = 8, Age 29–36 years, Male). Alkali-lysis method was adopted to isolate high molecular weight metagenomic DNA (Kumar et al., 2016). Qualitative and quantitative analysis of Metagenomic DNA was performed with agarose-gel electrophoresis and Qubit dsDNA HS Assay Kit (Invitrogen, United States) respectively.

### Small Subunit rRNA Gene Analysis

The V1 to V4 region of the SSU rRNA gene was amplified from the metagenomic DNA using region-specific primers (Morowitz et al., 2011). The amplified product was sequenced with Roche 454 GS FLX + using GS FLX Titanium XL^+^ Sequencing Kit (Gupta et al., 2017). Quantitative Insights into Microbial Ecology (QIIME) 1.9.1 pipeline was implemented for quality filtering, OTU picking, taxonomic assignment, alpha and beta diversity analysis (Kapono et al., 2018).

### Metagenome Sequencing and Sequence Analysis

The metagenomic DNA was sequenced with MiSeq Next-Generation Sequencing (NGS) platform with paired-end sequencing chemistry using MiSeq Reagent Kit v3 (600-cycle) (Illumina, United States). The reads were preprocessed and uploaded into the Metagenome Rapid Annotation using Subsystem Technology (MG-RAST) server 4.0.3 (Meyer et al., 2008). The metagenome sequence dataset was quality filtered (Denoising and normalization with DynamicTrim, removal of host DNA sequences with Bowtie2) and processed for the identification of rRNA gene features (by rRNA genecalling) and protein features (with genecalling using cutoff similarity% > 70%). Potential ribosomal RNA genes were clustered with CD-HIT and checked for their homologs in the Greengene database (*e*-value <10^–5^, sequence similarity < 60% and word size > 15 bp). FragGeneScan 1.3.1 was employed for the identification of all putative protein-coding features (Rho et al., 2010). Predicted protein features were clustered (90% identity) and processed for similarity search using BLAT (BLAST-Like Alignment Tool) algorithm (Kent, 2002) against the M5NR protein database, RefSeq database (O’Leary et al., 2016), COG database (Database of Clusters of Orthologous Groups of proteins) (Tatusov, 2000) with stringent search parameters (*e*-value <10^–5^, minimum identity < 60%). Protein feature abundance, Lowest Common Ancestor (LCA) abundance profile, and Data source abundance profile were used to predict taxonomic and functional affiliation of the predicated protein features (Milanese et al., 2019).

### Identification and Mapping of Features Associated With Vanillin Catabolism

Functional annotation of predicted protein features was performed by searching homologs in the Subsystems database (Overbeek, 2005) using stringent search parameters (*e*-value < 10^–5^, minimum identity < 60%, word size > 15). Vanillin catabolic features were manually curated from the annotated protein features and mapped on to the metabolic pathway. Sequence (both gene and protein) of the potential vanillin catabolic features were extracted from the metagenomic dataset and used for phylogenetic characterization using KAIJU webserver 1.7.3 (Menzel et al., 2016).

### Comparative Metagenome Analysis

To explore the omnipresence of the vanillin catabolic features across gender, age, geographical location, the human gut metagenomic datasets of the United States, Sweden, Venezuela, Japan, and Malaysia were used for comparative analysis (Supplementary Table S1). All these metagenomic datasets were processed with the MG-RAST server. Predicted RNA features were clustered and checked for their homologs in the Greengene database (*e*-value <10^–5^, sequence similarity < 60% and word size > 15 bp). Predicted protein features were clustered and searched for their homologs in the RefSeq database (O’Leary et al., 2016), COG database (Database of Clusters of Orthologous Groups of proteins) (Tatusov, 2000) (*e*-value <10^–5^, minimum identity < 60%). Functional annotation of the predicated protein features was performed after searching homologs (*e*-value < 10^–5^, minimum identity < 60%, word size > 15) in the Subsystems database. Vanillin catabolic features were manually curated from each dataset. Each search output was normalized before the Pearson correlation analysis, Principle Component Analysis (PCA), and heatmap generation (Koch et al., 2018).

### Functional Assessment of Vanillin Catabolism

Human fecal suspensions (*n* = 8) (500 mg/ml in phosphate buffer saline pH 7.4) were used for the purification of the microbial pellet (Kumar et al., 2016). The microbial pellet was incubated in vanillin solution (5 mM) at 37°C. A control sample without the vanillin solution was also incubated at 37°C simultaneously. The samples were withdrawn in small fractions at various intervals of time (0, 0.5, 2, 4, 6, 12, and 24 h). Samples were centrifuged at 13,000 rev min^–1^ for 2 min. Samples were quenched and processed for extraction of metabolites (Supplementary Method SM1). A 400 μl of acetonitrile was added to the metabolite fraction and centrifuged at 5,000 rev min^–1^ for 10 min. The supernatant was collected and loaded in autosampler for injection in LC-MS analysis. High-performance liquid chromatography coupled to quadrupole-time of flight mass spectrometry (HPLC/Q-TOF MS), possessing an Exion LC system integrated with X-500 QTOF (SCIEX Technology, United States) was used to obtain the metabolic profiles in the filtered supernatant (Supplementary Method SM2). Both negative and positive modes of electrospray ionization were used to capture the metabolic profile. LC-MS spectra were analyzed with SCIEX OS software 1.4 using ALL in One HR-MS/MS spectral library (SCIEX, United States) using an untargeted metabolic mapping workflow using default parameters.

## Results

### SSU rRNA Gene Amplicons Based Human Gut Microbiome Composition

**FIGURE 1:**
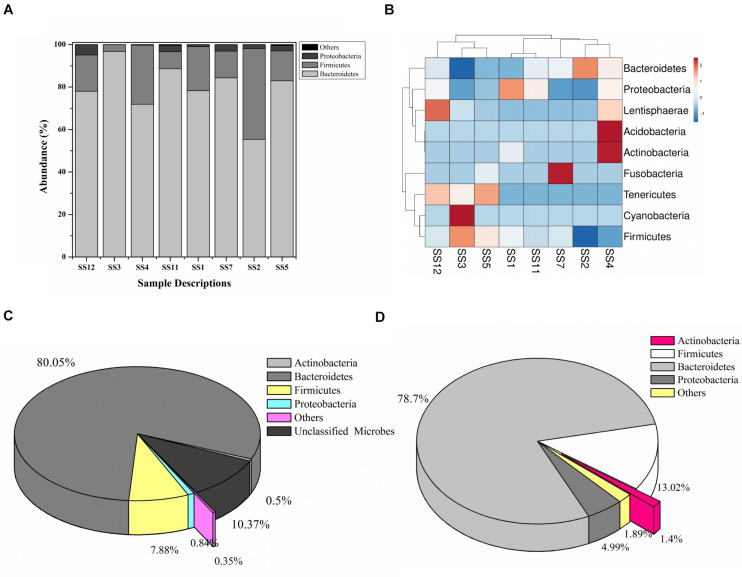
Microbiome composition based on SSU rRNA gene analysis, rRNA features, and protein features. Phylogenetic distribution of SSU rRNA genes **(A)** and their interrelationship among samples **(B)**. Phylogenetic distribution of human gut metagenomic rRNA features **(C)** and protein features **(D)**.

A total of 208,726 sequence reads were obtained after sequencing the SSU rRNA amplicons (V1–V4 region) from human stool metagenomic DNA. The removal of ambiguous sequences, quality (>Q30), and chimeric sequences, resulted in a total of 149,342 high quality reads for downstream analysis. *De novo* clustering of all sequences with QIIME 1.9.1 resulted in a total of 1,453 OTUs. Microbial diversity analysis of SSU rRNA sequences identified sequences corresponding to a total of 11 microbial phyla. Among all, the majority of sequences were of Bacteroidetes (80.18 ± 11.6%), Firmicutes (17.8 ± 11.67%), and Proteobacteria (1.85 ± 1.6%) lineages (Figures 1A,B). Additionally, representation of Actinobacteria, Acidobacteria, Chloroflexi, Fusobacteria, Lentisphaerae, Tenericutes, and Verrucomicrobia was also observed at lower proportions (Figures 1A,B). Within Bacteroidetes, majority of SSU rRNA sequences (90.16 ± 8.26%) were found affiliated to *Prevotella* sp., while *Ruminococcaceae* (40.82 ± 23.1%) and *Lachnospiraceae* (31.05 ± 17.85%) were found to be predominant among Firmicutes (Supplementary Figure S1).

### Taxonomic Characterization of the Human Gut Metagenomic Dataset

A total of 20,136,917 sequences were generated after shotgun sequencing of the human gut metagenomic DNA with the MiSeq paired-end sequencing chemistry (Illumina, United States) (Supplementary Table S2). The151554 sequences were identified to possess ribosomal RNA features. The majority of these ribosomal features were phylogenetically affiliated with the bacterial clade (99.98 ± 0.01%), whereas 0.01 ± 0.008% remained unclassified. Amidst bacterial clade, rRNA features shared homology with rRNA gene sequences of Bacteroidetes, Firmicutes, Proteobacteria, Actinobacteria, Verrucomicrobia, Cyanobacteria, Tenericutes, Chlorobi, Synergistetes, Planctomycetes, and Spirochetes (Figure 1C). Among these groups, Bacteroidetes were predominant (66.11 ± 24.67%) followed by Firmicutes (17.47 ± 16.95%), and Proteobacteria (2.86 ± 2.47%). The ribosomal features based gut microbiome analysis shared a good correlation (*R* = 0.9916) with the outcome of the SSU rRNA gene-based microbial diversity analysis. This highlights the significant concordance among the microbial diversity observed after whole metagenome sequencing and targeted V1–V4 region of 16S rRNA gene sequencing.

RefSeq database search analyses indicated taxonomic affiliation within bacteria (99.62 ± 0.1%), archaea (0.21 ± 0.06%), eukaryota (0.14 ± 0.03%), and viruses (0.035 ± 0.01%). These protein features showed similar phylogenetic distribution as observed after analysis of SSU rRNA genes and rRNA features (Figure 1D and Supplementary Table S3). Despite of a slightly higher diversity of the encoded protein features, a good correlation was observed among phylogenetic affiliation of encoded protein features and SSU rRNA gene-based diversity analysis (*R* = 0.975).

### Functional Characterization of the Human Gut Metagenomic Dataset

A total of 2,581 clusters of orthologous groups (COGs) have been observed with a role in various cellular physiological processes. Of this, 48.93 ± 2.30% of orthologous features were found associated with cellular metabolism, while 20.10 ± 0.17% were involved in cellular processes and signaling. A slightly lower proportion (19.01 ± 2.07%) was found associated with information storage and processing, while remaining (11.94 ± 0.16%) were poorly characterized proteins. Among cellular metabolism-associated COGs, the majority of proteins were associated with carbohydrate transport and metabolism (30.18 ± 0.69%) and amino acid transport and metabolism (23.53 ± 0.17%). We observed a very low percentage of COGs (3.11 ± 0.11% and 0.57 ± 0.03%) associated with defense and secondary metabolism (Supplementary Table S4). The human gut is considered a more stable ecosystem than its environmental counterparts (soil, water, etc.). Hence human gut microbes were not subjected to challenging environmental conditions (variations in temperature, pH, water contents, scarcity of nutrients, etc.) (Tian et al., 2017). Human gut microbiome evolution might have allowed human gut microbes to perform genetic restructuring toward gut conditions for better harvesting of energy and nutrients for their growth and proliferation (Hooper, 2001).

### Vanillin Catabolism Genetic Machinery of the Human Gut Microbiome

To date there is no scientific evidence of vanillin catabolism among human gut microbes; however, its catabolism has been studied in various free-living microbes like *Rhodococcus jostii* RHA1 (Chen et al., 2012), *Pseudomonas putida* (Plaggenborg et al., 2003), *Sphingomona spaucimobilis* SYK-6 (Masai et al., 2007), and *Streptomyces* sp. NL15-2K (Nishimura et al., 2018). Vanillin is generally metabolized in two phases; vanillin is catabolized into protocatechuate by two-step process catalyzed by vanillin dehydrogenase (LigV) and vanillate O-demethylase (LigM) in the first phase (Kamimura et al., 2017). Phase II includes bioconversion of protocatechuate through a central aromatic intermediate metabolic pathway into TCA cycle intermediates (Grant and Patel, 1969; Romero-Steiner et al., 1994; Parke, 1995).

**FIGURE 2:**
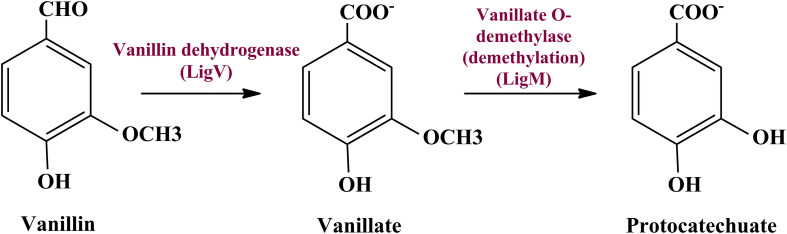
The delineated phase I of the vanillin catabolic pathway. Vanillin dehydrogenase (VanD) and vanillate O-demethylase (VanM) metabolize vanillin into protocatechuate.

Manual curation of the human gut metagenome dataset identifies protein features and their respective genes potentially associated with vanillin metabolism (Table 1). Homologs of the decoded genes are part of *lig*, *pca*, and *cat* operons. Encoded products of vanillin membrane transporter gene (*vanK*), *lig* genes (*ligV* and *ligM*) could perform cellular transport and catabolism of vanillin into protocatechuate, respectively (Figure 2). Identified vanillin membrane transporter (VanK) was found to be a member of the aromatic acid/H + symport family MFS transporter involved in the transport of aromatic compounds across cytoplasmic membranes (Chaudhry et al., 2007). The *lig* operon genes *ligV*, and *ligM* encode putative vanillin dehydrogenase (LigV) and putative vanillin-O-demethylase (LigM). Homologs of these proteins catalyze the conservation of vanillin to vanillic acid and vanillic acid to protocatechuate (Chen et al., 2012). Putative vanillin dehydrogenase (LigV) sequence was found to harbor a ALDH_VaniDH_like (*Pseudomonas putida* vanillin dehydrogenase-like) conserved domain (cd07150) characterized for NAD(P)^+^-dependent dehydrogenase activity against vanillin. Additionally, putative LigV features shared a good homology of (65–90%) with their characterized homolog of *Pseudomonas putida* KT2440 indicating its activity as a vanillin dehydrogenase. The sequences of putative LigM were found to possess START/RHO_alpha_C/PITP/Bet_v1/CoxG/CalC (SRPBCC) ligand-binding domain. The SRPBCC domain was characterized by Rieske-type non-heme iron aromatic ring-hydroxylating oxygenases. Additionally, putative LigM shared a very good homology of 98 and 96% with vanillin-O-demethylase of *Enterobacter hormaechei* and *Klebsiella pneumonia* respectively. Presence of a domain for Rieske-type non-heme iron aromatic ring-hydroxylating oxygenases, and high identity with vanillin-O-demethylase indicates its potential activity as vanillin-O-demethylase to convert vanillic acid into protocatechuate. Phylogenetic characterization indicates that the VanK, LigV, and LigM shared homology with the proteins belonging to the *Azotobacter vinelandii*, *Azoarcus* sp., *Enterobacter hormaechei, Klebsiella pneumonia, Pseudomonas putida*, *Rhodopseudomonas palustris*, and *Serratia proteamaculans* indicating their origin from the proteobacterial clade.

**TABLE 1:** Human gut microbiome protein features mapped for vanillin catabolism.

Vanillin metabolism	Protein features	Representative features
Phase I	Vanillin dehydrogenase, LigV (EC 1.2.1.65)	4
(Vanillin biotransformation)	Vannilate transporter, VanK	5
	Vanillate O-demethylase, LigM (EC 1.14.13.82)	21
Phase II	Protocatechuate 3,4-dioxygenase alpha chain, PcaG (EC 1.13.11.3)	4
(Ring fission)	Protocatechuate 3,4-dioxygenase beta chain, PcaH (EC 1.13.11.3)	12
	3-carboxy-cis,cis-muconate cycloisomerase, PcaB (EC 5.5.1.2)	11
	4-carboxymuconolactone decarboxylase, PcaC (EC 4.1.1.44)	553
	Beta-ketoadipate enol-lactone hydrolase, PcaD (EC 3.1.1.24) or 3-oxoadipate enol-lactonase, PcaD (EC 3.1.1.24)	2
	3-oxoadipate CoA-transferase subunit A, PcaI (EC 2.8.3.6)	7
	3-oxoadipate CoA-transferase subunit B, PcaJ (EC 2.8.3.6)	1
	Beta-ketoadipyl CoA thiolase, PcaF (EC 2.3.1.16)	5
	Pca operon regulatory protein, PcaR	19
	Succinyl-CoA:3-ketoacid-coenzyme A transferase subunit A, ScoA (EC 2.8.3.5)	2
	4-hydroxybenzoate transporter, PcaK	15
	Catechol 1,2-dioxygenase, CatA (EC 1.13.11.1)	7
	Muconate cycloisomerase, CatB (EC 5.5.1.1)	13
	Muconolactone isomerase, CatC (EC 5.3.3.4)	1

**FIGURE 3:**
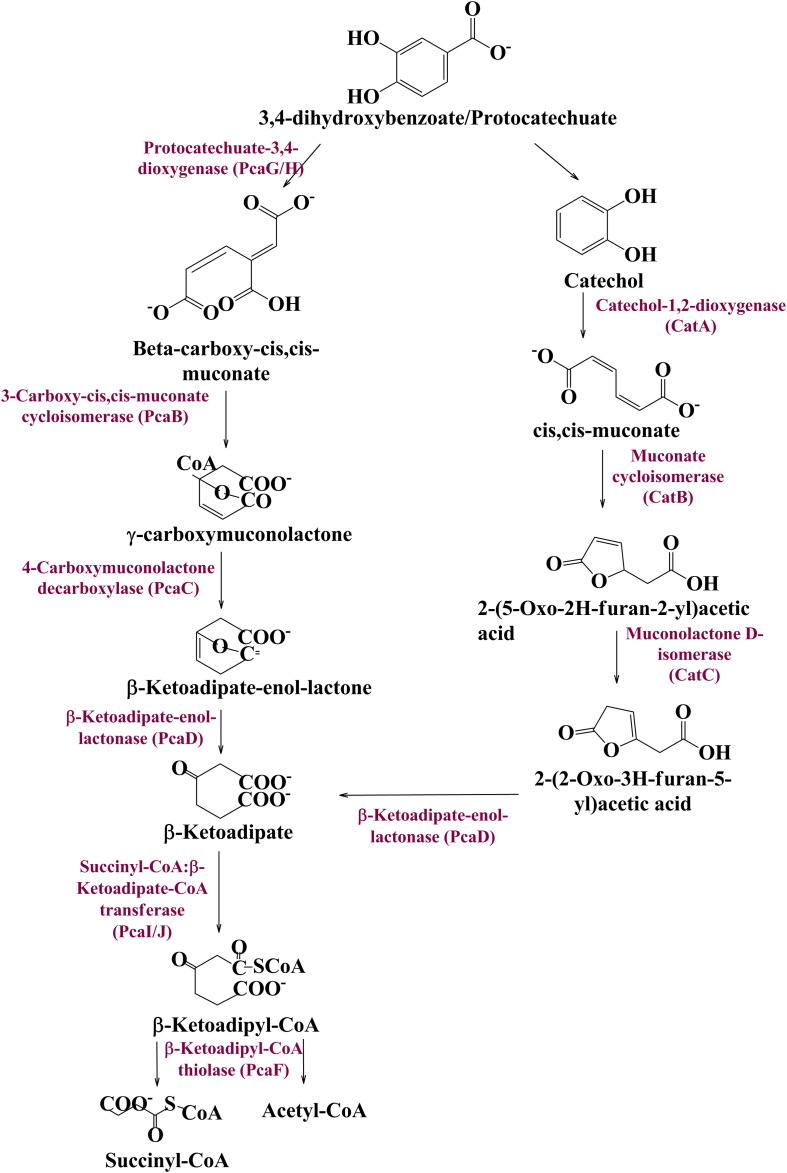
The delineated phase II of the vanillin catabolic pathway. Protocatechuate could be metabolized either directly into TCA cycle intermediates or through catechol mediated ortho ring cleavage.

Metagenomic exploration identifies genes associated with the catechol branch of the beta-ketoadipate pathway and the protocatechuate branch of the beta-ketoadipate pathway (Table 1). The *pcaIJFBDKCHG* operon is well-established as genetic machinery to catabolize protocatechuate to TCA cycle intermediates through the beta-ketoadipate pathway (Gerischer et al., 1998). Manual curation of the current metagenomics dataset indicates several genes sharing homology with *pcaB*, *pcaC*, *pcaD*, *pcaG/H, pcaI, pcaJ, pcaK, pcaF*, and *pcaR* (Table 1). Encoded proteins of these genes systematically catabolize protocatechuate into succinate and acetyl CoA (Figure 3). These putative proteins share homology with the proteins associated with Proteobacteria, Firmicutes, and FCB group microbes (*Acidovorax* sp., *Bacteroides fragilis*, *Dethiosulfovibrio peptidovorans*, *Eggerthella lenta*, *Escherichia coli, Eubacterium rectale*, *Klebsiella pneumoniae, Lactobacillus acidophilus*, *Lactobacillus brevis*, *Lactobacillus gasseri*, *Lactobacillus reuteri*, *Leuconostoc mesenteroides*, *Methanosarcina barkeri*, *Marinomonas*, *Pediococcus pentosaceus*, *Pseudomonas syringae*, *Rhodospirillum centenum*, *Ruegeria pomeroyi*, and *Streptococcus mutans*).

Catechol mediated protocatechuate metabolism is an alternative catabolic pathway (Grant and Patel, 1969), catabolized by Catechol-1,2-dioxygenase, Muconate cycloisomerase, and Muconolactone D-Isomerase (Figure 3) encoded by *catA*, *catB*, and *catC* genes of cat operon, respectively. In-depth human gut metagenome analysis showed several protein features homologous to the CatA, CatB, and CatC proteins (Table 1). Human gut metagenomic putative Catechol 1,2-dioxygenase (*catA*) were found phylogenetically affiliated with proteins of the *Klebsiella pneumoniae*, while the putative Muconate cycloisomerase (*catB*) shared homology with the proteins of *Bacteroides vulgatus, Dokdonia donghaensis*, and *Clostridium difficile* origin. Muconolactone isomerase (*catC*) shared homology with Crenarchaeota (*Sulfolobus tokodaii*) originated proteins.

**FIGURE 4:**
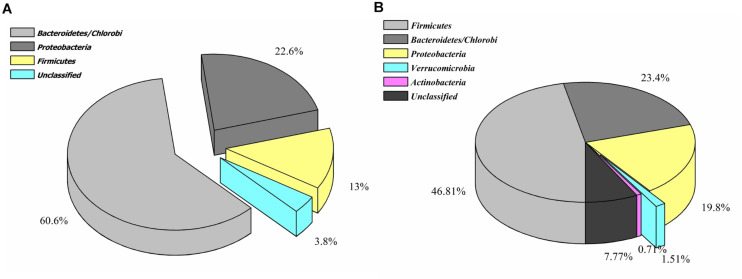
Phylogenetic affiliation of the features associated with catechol **(A)** and protocatechuate **(B)** catabolism.

Contrary to the vanillin catabolic gene clusters, *pca*, and *cat* operon encoded proteins shared homology with diversified microbial groups (Figure 4). These observations indicate that vanillin metabolism might be either carried out in a synergistic way or there is a division of labor where only one microbial group is assigned to do this job.

### Comparative Metagenomic Analysis

Current human gut metagenomic datasets were compared with other gut metagenome datasets (Supplementary Table S1) to assess its similarity and uniqueness. Selected gut metagenomic datasets represented the individual of diverse geographical locations (United States, Sweden, Venezuela, Japan, and Malaysia), varied age group (infants, children, teenagers, and adults) (Yatsunenko et al., 2012), gender (male and female). Presence of vanillin catabolic features among this varied representation would confirm their omnipresence.

Ribosomal features of the current metagenomic dataset showed a good correlation with ribosomal features identified in other metagenomic datasets at the domain level (*R* > 0.99), however, it is drastically reduced at lower taxonomic levels (Supplementary Table S5). Principal component analysis (PCA) indicates a clustering of the current metagenomic dataset with the metagenomic dataset of Japan and Venezuela at the domain level (Supplementary Figure S2A); however, no clustering was observed at other taxonomic analysis (Supplementary Figures S2B,C). Heatmap indicates variable clustering of the current metagenomic dataset with a metagenomic dataset at different taxonomic levels (Supplementary Figure S3). These results cumulatively indicate that the gut metagenomic datasets are comparatively similar at a higher taxonomic level; however, phylogenetic variability enhances at lower taxonomic levels.

**FIGURE 5:**
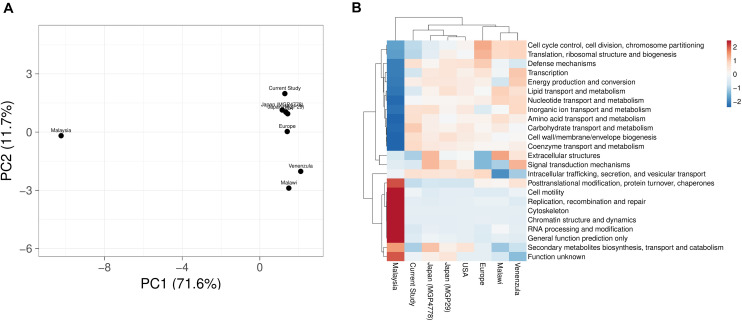
A comparison of the metabolic potential of the gut metagenomes across diversified populations. Principle component analysis **(A)** and heatmap **(B)** showing the relationship among various gut metagenomes.

COGs analysis of the metabolic features showed a very good correlation at level 1 (*R* > 0.99) and level 2 (*R* > 0.96, *R* > 0.98, *R* > 0.95, *R* > 0.9606, *R* > 0.98, and *R* > 0.95) of COG classification with the features identified in various metagenomic datasets (Malawi, Japan, Europe, United States, and Venezuela, respectively). However, a lower correlation (*R* = 0.45) was observed with Malaysian population gut metagenomic dataset at level 2 of COG classification. Principal component analysis (PCA) also makes a similar observation (Figure 5A). Heatmap of the subsystem database identified protein features showed clustering of the current metagenomic datasets with Malawi, Japan, Europe, United States, Venezuela in a group, while Malaysian metagenome was in the distant group (Figure 5B). Comparative functional profiling of metagenomic datasets indicates that despite taxonomic diversity, all the metagenomic dataset harbors similar functional profile.

**FIGURE 6:**
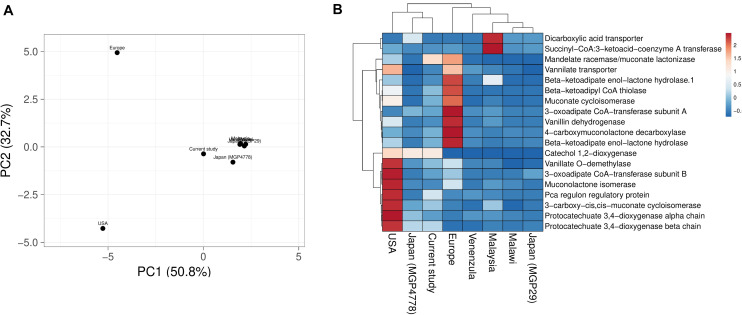
Vanillin catabolic features across various gut metagenomes. Principal component analysis **(A)** and heatmap **(B)** showing the relationship among various gut metagenomes.

Explorations of the metagenomic datasets for vanillin catabolic features indicate their omnipresence with differential abundance profile (Supplementary Table S6). Principal component analysis (PCA) and heatmap indicated clustering of Japan (MGP4778) and United States metagenomes with the current dataset (Figure 6). The omnipresence of vanillin catabolic features indicates the metabolic potential of the gut microbiome. However, their functioning needs to be assessed through functional assays.

### Validation of Vanillin Catabolism

Metabolomics profiling identified several metabolites associated with vanillin metabolism (Table 2). Significantly enriched metabolites were mapped to the proposed metabolic pathway confirming the functionality of the proposed vanillin metabolic pathway. LC-MS spectra analysis indicates that the vanillin was completely metabolized by human gut microbes within 24 h of incubation (Supplementary Figure S4). The disappearance of vanillin was negatively correlated with the appearance of the Protocatechuate (*r* = -0.987), Catechol (*r* = -0.978). These results established the protocatechuate and catechol-mediated vanillin catabolism among human gut microbes.

**TABLE 2:** List of statistically significant (*p* < 0.05) metabolites associated with vanillin metabolism from the microbial pellet incubated with 5 mM vanillin at 37°C for 24 h.

Metabolite	Rf value	m/z value	*p*-value
Protocatechuate	6.26	153.019	0.0315
Catechol	6.26	109.030	0.0470
Beta-carboxy-cis,cis-muconate	6.38	181.986	0.0001
Beta-ketoadipate	16.68	159.030	0.0339

## Discussion

The human microbiome is a specialized dynamic organ that plays a vital role in the maintenance of host physiology. The human gut microbiome complements with host machinery to metabolize a wide range of ingested foods (Yadav et al., 2018). Along with improving the metabolic capacity of the host, the gut microbes play a significant role in drug detoxification (Jourova et al., 2016) and xenobiotic metabolism (Clarke et al., 2019). Vanillin, a commonly used major food additive, possesses antimicrobial, antioxidative, and flavoring properties (Fitzgerald et al., 2004; Ngarmsak et al., 2006). Metabolism of vanillin was delineated in the human body (Muskiet and Groen, 1979) where the liver metabolized it as vanillic acid and finally it was excreted as a free metabolite or in conjugate form through the urogenital system (Sayavongsa et al., 2007). Though the human gut microbiome plays a key metabolic component in food digestion, the role of the human gut microbiome in vanillin metabolism has never been studied. Additionally, there is a lack of information about microbial survival strategies toward antimicrobial properties of vanillin. The present study encompasses an integrative approach wherein the human gut microbiome has been shown to delineate the process of vanillin catabolism to counter its antimicrobial properties.

The studied human gut microbiome is primarily composed of Bacteroidetes, Firmicutes, and Proteobacteria, while Actinobacteria and other microbial groups contribute less to the total gut bacterial diversity. Efforts to analyze the human gut microbial diversity in a similar population also revealed the matching outcome wherein Bacteroidetes is the predominant bacteria within the human gut (Human Microbiome Project Consortium, 2012; MetaHIT Consortium (additional members) et al., 2011). Additionally, *Prevotella* is the predominant microbial group within the Bacteroidetes and *Ruminococcaceae* and *Lachnospiraceae* among Firmicutes. These observations are in line with human gut microbial diversity studies highlighted by our group (Chauhan et al., 2018), as well as by other studies in Indian populations (Bhute et al., 2016). SSU rRNA gene amplification followed by their sequencing could introduce biases during the exploration of microbial diversity (Poussin et al., 2018). Hereby, the current study also assesses the microbial diversity through shotgun sequenced datasets that are considered more precise and accurate approach (Ranjan et al., 2016). The metagenomic diversity revealed through RNA and protein feature analysis indicated the abundance of Bacteroidetes followed by Firmicutes and Proteobacteria along with traces of archaea, viruses, fungi, and higher eukaryotes. These observations are in line with human gut microbial profiles generated from various global populations in the current study, as well as in other studies (MetaHIT Consortium (additional members) et al., 2011). Despite it, variability in microbial taxonomic distribution and abundance profile was observed across various populations. These variations could be attributed to diverse food habits, ethnicity, and lifestyle (Chauhan et al., 2018). Functional annotation of human gut metagenomic protein features shows an enrichment of the carbohydrate metabolism associated protein features. Harvesting energy from non-conventional carbohydrates might be allowing the human gut microbes to avoid any conflict with the host to meet their energy requirement (Flint et al., 2012). These interactions might be playing an active role in developing commensalism with the host toward the successful commensalism of the human gut microbiome (Kumar Mondal et al., 2017). In addition to the CAzyme associated protein features, a diverse array of the proteins associated with microbial growth and sustenance were identified (Kurokawa et al., 2007). This variability could be explained in terms of their evolutionary adaptations to meet up environmental requirements (Bradley and Pollard, 2017).

Protein features potentially associated with the vanillin metabolism were identified from the human gut metagenome dataset. Protein features like membrane-bound vanillin transport systems (VanK), vanillin dehydrogenase (ligV), and vanillin-O-demethylase (ligM) indicate the cellular process of vanillin metabolism in the human gut microbes. As characterized in *Acinetobacter* sp., VanK feature might be allowing the human gut microbes for the cellular transport of vanillin and Protocatechuate (D’Argenio et al., 1999). Similarly, ligV and ligM as characterized in *Sphingomonas paucimobilis* SYK-6, *Pseudomonas putida* (Abe et al., 2005; Masai et al., 2007) indicate vanillin catabolism into protocatechuate. The current metagenomic dataset also represents the presence of almost all genes associated with *pca* operon. These gene clusters were characterized for protocatechuate metabolism to TCA cycle intermediates through the beta-ketoadipate pathway in the free-living microbes (Iwagami et al., 2000). Similarly, cat operon genes *catA* (Catechol-1,2-dioxygenase), *catB* (Muconate cycloisomerase), and *catC* (Muconolactone-D-isomerase) have been characterized in free-living microbes for catabolizing catechol through the beta-ketoadipate intermediate of the central aromatic intermediate metabolic pathway (Cámara et al., 2007). Metabolic mapping of all these human gut microbiome protein features indicates a probable pathway for vanillin metabolism.

Presences of the vanillin catabolic features across diverse populations with varied age, sex, and geography indicates their omnipresence. The gut metagenome of Europe, United States, Venezuela and Malaysian population showed a relative higher enrichment of vanillin specific (Phase I) catabolic features in comparison to Japan and Malawi population. This varied enrichment of vanillin catabolic features could be an outcome of differential exposure to the vanillin supplemented foods; however the lack of vanillin consumption dataset across the global population limits the validation of this hypothesis.

However, the presence of genetic features does not confirm their functioning (Langille, 2018), hereby a translation approach needs to be implemented to confirm their function in the gut microbes (Zhang et al., 2019). Metabolomics approach was used for validating the functioning of vanillin catabolic features. It has shown statistical enrichment of metabolites associated with the proposed pathway. Additionally, a reciprocal relationship among the disappearance of vanillin with enrichment of protocatechuate and catechol also confirms the vanillin protocatechuate-mediated vanillin metabolism.

Phylogenetic affiliation analysis of protein features indicates that the vanillin specific catabolic features were selectively enriched in gammaproteobacteria group microbes, while protein features associated with protocatechuate metabolism showed omnipresence in the human gut microbial groups belonging to Firmicutes, Bacteroidetes, Proteobacteria, and Actinobacteria. This segregated distribution indicates either vanillin catabolism is the only function in proteobacteria microbial groups or there is a division of labor as well as stratified functioning in the gut ecosystem (Sichertz, 2011). Additionally, the majority of these identified genetic features shared homology with genetic features of the free-living microbes (Kumar Mondal et al., 2017). It indicates a possible transfer of the genes in the human gut microbiome through horizontal gene transfer for a better adaptation within the human gut environment (Lerner et al., 2017).

Though the current study helps to explain the mechanism of vanillin catabolism, it raises the scientific query as to whether the host microbiome is already enriched with such metabolic machinery or exposure of these xenobiotics has modulated the human gut microbes to evolve (Maurice et al., 2013). Progressive evolution is a prominent feature of the microbes, which allows them to adapt and colonize in extreme environments (Li et al., 2014). It strongly favors the possibility that the continuous exposure of a xenobiotic has put an evolutionary pressure on the human gut microbes to enrich their genetic machinery to either protect from their antimicrobial nature (Sarmiento et al., 2019) or to harness energy from it (Dantas et al., 2008).

The current study is the first study to catalog human gut microbial gene clusters and the protein features involved in vanillin catabolism. Additionally, the current study has used the strength of multi-omics approach to rationalize the role of human gut microbiome composition in vanillin catabolism. This study strengthens the hypothesis of gut microbiome evolution with respect to dietary composition, as well as explains how microbes accustom to the changing gut environment for successful colonization.

## Data Availability Statement

Metagenomic DNA sequences can be found in MG-RAST server with “ab6d97f3c66d676d343633383135322e33id and 9a02e1b94c6d676d343633383135332e33” (https://www.mg-rast.org/mgmain.html?mgpage=token&token=aujodAvGtxRPyZ 11oYYVeBW4Zno1J0LQuAT66Q6XSCWmKTBtow).

## Ethics Statement

The studies involving human participants were reviewed and approved by the M. D. University, Rohtak, Haryana, India. The patients/participants provided their written informed consent to participate in this study.

## Author Contributions

NC designed the study and experiments and analyzed the data. NC, MY, and RP wrote the manuscript. MY carried out the experiments. All authors edited the manuscript and approved the final draft of the manuscript.

## Conflict of Interest

The authors declare that the research was conducted in the absence of any commercial or financial relationships that could be construed as a potential conflict of interest.

## Supplementary Material

Supplementary Figure 1 Phylogenetic affiliation of SSU rRNA sequences derived from human feces metagenomic DNA. Taxonomic of SSU rRNA sequences affiliated with Bacteroidetes **(A)** and Firmicutes **(B)**.

Supplementary Figure 2 Principle component analysis plots at different taxonomic levels [domain **(A)**, phylum **(B)**, and class **(C)** taxonomic levels].

Supplementary Figure 3 Heatmap showing correlation of representative ribosomal features among metagenomic datasets at different taxonomic levels [domain **(A)**, phylum **(B)**, and class **(C)** taxonomic levels].

Supplementary Figure 4 The functional assessment of vanillin catabolism using LC-MS analysis.

Supplementary Table 1 Human gut metagenome datasets used for comparative analysis.

Supplementary Table 2 Statistics of the current human gut metagenome dataset.

Supplementary Table 3 Relative abundance of the sodium benzoate catabolic features across various gut metagenomes.

Supplementary Table 4 Abundance profile of the Cluster of orthologous groups (COGs) identified in current metagenomic datasets.

Supplementary Table 5 Correlation among the ribosomal features of the current metagenomic dataset with other datasets at different taxonomic levels.

Supplementary Table 6 Abundance profile of vanillin catabolic features across gut metagenome datasets.

Supplementary Method 1 Sample processing and chromatographic separation.

Supplementary Method 2 Mass spectrometric detection of metabolites.
