# Supplementary material for: Catabolic Machinery of the Human Gut Microbes Bestow Resilience Against Vanillin Antimicrobial Nature
Source: Front Microbiol. 2020 Oct 16;11:588545. doi: 10.3389/fmicb.2020.588545 (PMC7605359; doi:10.3389/fmicb.2020.588545)

Catabolic machinery of the human gut microbes bestow resilience against vanillin antimicrobial nature

Monika Yadav[1], Rajesh Pandey[2† *], and Nar Singh Chauhan[1*]

[1]Department of Biochemistry, Maharshi Dayanand University, Rohtak, Haryana, India

[2]Genomics and Molecular Medicine, CSIR-Institute of Genomics and Integrative Biology (CSIR-IGIB) Delhi-110007, India

*Corresponding author

Nar Singh Chauhan (nschauhan@mdurohtak.ac.in)

†Co- Corresponding author

Rajesh Pandey (rajeshp@igib.res.in)

(A) Supplementary methods

Supplementary method SM1: Sample processing and chromatographic separation

Quenching of the samples was performed by incubating with 60% aqueous methanol solution (-48 °C). Samples were centrifuged immediately at 16060*g* for 10 min at -10 °C followed by snap-freezing in liquid nitrogen. Metabolites were extracted with methanol by freeze-thaw cycling. Samples were centrifuged immediately at 16060*g* for 10 min at -10 °C to collect the supernatant followed by its storage at -86ºC. An additional volume of 500 µL of 100% methanol (-48 °C) was added to the pellet followed by freeze-thaw cycling. Samples were centrifuged again at 16060*g* for 10 min at -10 °C to collect the supernatant. Both supernatants (first and second cycle) were pooled and stored at -86ºC for further analysis. A 400µl acetonitrile (HPLC grade) was added in each vial followed by centrifugation at 5000 rev min^{-1} for 10 minutes and the supernatant was collected. Untargeted metabolic profiling was performed with Exion LC system integrated AB SCIEX X500B QTOF mass spectrometer (SCIEX, USA). ExionLC™ System fitted with an Agilent PLRP-S column (2.1 mm×50 mm, 300 Å, 5 µm) was employed for chromatographic separation of metabolites. Chromatography was carried out at 80°C using acetonitrile gradient (Table SM1). The column was equilibrated with the mobile phase for 30 min prior to the sample injection. Sample injection volume was 10.0 µL, and the total run time was 20 min per sample.

Table SM1. Liquid chromatographic conditions used in this study

Time (min)	Flow (mL min^{-1})	0.1% formic acid in water (%)	0.1% formic acid in acetonitrile (%)
4.00	0.6000	95.0	5.0
8.00	0.6000	50.0	50.0
10.00	0.6000	50.0	50.0
14.00	0.6000	5.0	95.0
16.00	0.6000	5.0	95.0
18.00	0.6000	95.0	5.0
20.00	0.6000	95.0	5.0

Supplementary method SM2: Mass spectrometric detection of metabolites

AB SCIEXX500B QTOF mass spectrometer with a Turbo V Ion Source fitted with a Twin Sprayer ESI Probe was operated in the ESI- & ESI+ mode for data acquisition. Mass spectrophotometer instrument experimental conditions are listed in Table 2. Data was captured with SCIEX OS 1.4.

Table SM2. Mass spectrophotometer instrument conditions in the current study

Parameter	ESI +ve mode	ESI -ve mode
Gas 1	50 psi	50 psi
Gas 2	60 psi	50 psi
Curtain gas	35 psi	35 psi
Temperature	550 °C	550 °C
Ion Spray Voltage	5500 V	-4500 V
Time Bin to Sums	4	4
Accumulation Time	0.25s	0.25s
TOF Start Mass	100 da	100 da
TOF Stop Mass	2000 da	2000 da
Declustering Potential	80V	-80V
Collision Energy	10V	-10V

(B) Supplementary Tables

Supplementary Table S1. Human gut metagenome datasets used for comparative analysis.

Sr No	Origin of the Metagenomic Dataset	Nature of Dataset	Datasets	Accession ID
1	USA	Unassembled reads	59	MGP98 (www.mg-rast.org)
2	Sweden	Unassembled reads	80	MGP13068 (www.mg-rast.org)
3	Japan	Unassembled reads	13	MGP29 (www.mg-rast.org)
4	Japan	Unassembled reads	13	MGP4778 (www.mg-rast.org)
5	Malawi	Unassembled reads	19	MGP98 (www.mg-rast.org)
6	Venezuela	Unassembled reads	21	MGP98 (www.mg-rast.org)
7	Malaysia	Unassembled reads	8	MGP5712 (www.mg-rast.org)

Supplementary Table S2. Statistics of the current human gut metagenome dataset.

Total bp Count	12,068,464,868 bp
Artificial Duplicate Reads	10117308
Basepairs Count after QC	5474023416 bp
Sequences Count after QC	27037796
Mean Sequence Length after QC	403 ± 134 bp
Mean GC percent after QC	48 ± 7 %
Predicted Protein Features after QC	12,077,035

Supplementary Table S3. Phylogenetic affiliation of the protein features identified in the current dataset.

Phylum	Abundance (%)
Acidobacteria	0.00770638
Actinobacteria	0.139525335
Apicomplexa	0.000312677
Aquificae	0.00222932
Arthropoda	0.000586317
Ascomycota	0.00237387
Bacillariophyta	0.000185132
Bacteroidetes	7.869747336
Basidiomycota	0.00027364
CandidatusPoribacteria	0.000137593
Chlamydiae	0.001310228
Chlorobi	0.014421781
Chloroflexi	0.006810865
Chlorophyta	0.000539164
Chordata	0.001554494
Chrysiogenetes	0.000437516
Cnidaria	0.000346302
Crenarchaeota	0.000703426
Cyanobacteria	0.0131417
Deferribacteres	0.001990464
Deinococcus-Thermus	0.003054106
Dictyoglomi	0.00182311
Echinodermata	5.95207E-05
Elusimicrobia	0.001265007
Euglenida	7.72996E-07
Euryarchaeota	0.016913919
Fibrobacteres	0.013110393

Firmicutes	1.302473382
Fusobacteria	0.016145947
Gemmatimonadetes	0.000269389
Hemichordata	7.34346E-06
Korarchaeota	3.40118E-05
Lentisphaerae	0.007816919
Microsporidia	4.21283E-05
Nanoarchaeota	7.72996E-07
Nematoda	0.000149575
Nitrospirae	0.001024219
Phaeophyceae	1.15949E-06
Placozoa	5.21772E-05
Planctomycetes	0.002410974
Platyhelminthes	1.04354E-05
Proteobacteria	0.499405458
Spirochaetes	0.014380425
Streptophyta	0.001115433
Synergistetes	0.005085152
Tenericutes	0.002952844
Thaumarchaeota	7.30481E-05
Thermotogae	0.006441373
Verrucomicrobia	0.031747319
Unclassified (derived from Bacteria)	0.000880829
Unclassified (derived from Eukaryota)	0.004035424
Unclassified (derived from Viruses)	0.002742589
Unclassified (derived from other sequences)	0.000253543

Supplementary Table S4. Abundance profile of the Cluster of orthologous groups (COGs) identified in current metagenomic datasets.

COGs Clustering (Level-1)	COGs Clustering (Level-2)	Abundance (%)
Cellular processes and signaling	Cell cycle control, cell division, chromosome partitioning	0.852646239
Cellular processes and signaling	Cell motility	0.176844244
Cellular processes and signaling	Cell wall/membrane/envelope biogenesis	7.615568383
Cellular processes and signaling	Cytoskeleton	0.001857352
Cellular processes and signaling	Defense mechanisms	3.175036218
Cellular processes and signaling	Extracellular structures	0.0075512
Cellular processes and signaling	Intracellular trafficking, secretion, and vesicular transport	2.321446079
Cellular processes and signaling	Posttranslational modification, protein turnover, chaperones	3.506771417
Cellular processes and signaling	Signal transduction mechanisms	2.368367047
Information storage and processing	Chromatin structure and dynamics	0.001065694
Information storage and processing	RNA processing and modification	0.004415016
Information storage and processing	Replication, recombination and repair	7.810803454
Information storage and processing	Transcription	3.277860428
Information storage and processing	Translation, ribosomal structure and biogenesis	6.785575746
Metabolism	Amino acid transport and metabolism	11.77344815
Metabolism	Carbohydrate transport and metabolism	15.33149466
Metabolism	Coenzyme transport and metabolism	4.338134232
Metabolism	Energy production and conversion	6.497838469
Metabolism	Inorganic ion transport and metabolism	5.59132903
Metabolism	Lipid transport and metabolism	2.394430868
Metabolism	Nucleotide transport and metabolism	3.668056534
Metabolism	Secondary metabolites biosynthesis, transport and catabolism	0.583969654
Poorly characterized	Function unknown	0.604522317
Poorly characterized	General function prediction only	11.31096757

Supplementary Table S5. Correlation among the ribosomal features of the current metagenomic dataset with other datasets at different taxonomic levels.

Taxonomic Level	Malawi	Japan (MGP4778)	Japan2 (MGP 29)	Malaysia	Europe	USA	Venezuela
Domain	0.999999667	0.999998	1	1	0.999999	0.999999	1
Phylum	0.434643202	0.38289	0.268309	-0.0248	0.316315	0.379972	0.509737
Class	0.789851539	0.64336	0.663018	0.912729	0.868018	0.780327	0.781199

Supplementary Table S6. Abundance profile of vanillin catabolic features across gut metagenome datasets.

**Vanillin Catabolic Feature**	**Malawi**	**Japan (MGP4778)**	**Japan (MGP29)**	**Malaysia**	**Europe**	**USA**	**Venezuela**
Protocatechuate 3,4-dioxygenase alpha chain	1.063829787	6.08108	0	2.45902	0	4.348	1.8867925
Protocatechuate 3,4-dioxygenase beta chain	1.063829787	8.10811	3.3898	1.63934	0	3.804	0
3-carboxy-cis,cis-muconate cycloisomerase	0	4.72973	3.3898	9.01639	0.05681	3.895	0.6289308
4-carboxymuconolactone decarboxylase	86.17021277	45.9459	71.186	1.63934	84.3969	45.29	74.213836
3-oxoadipate CoA-transferase subunit A	0	7.43243	0	4.09836	1.45806	0.091	1.8867925
3-oxoadipate CoA-transferase subunit B	0	0	1.6949	0	0.03787	0.906	0
Beta-ketoadipateenol-lactone hydrolase	1.063829787	0	5.0847	0	2.49953	2.899	2.5157233
Beta-ketoadipyl CoA thiolase	0	0	0	0	0.35978	0.815	0
Pcaregulon regulatory protein	0	0	0	1.63934	0.03787	5.616	2.5157233
Beta-ketoadipateenol-lactone hydrolase	0	0	0	8.19672	0.43552	0.543	0.6289308
Dicarboxylic acid transporter	1.063829787	2.7027	1.6949	9.83607	0	0	0
Catechol 1,2-dioxygenase	0	4.72973	0	0	0	0.725	0
Muconate cycloisomerase	1.063829787	0.67568	3.3898	3.27869	1.17402	3.442	0
Muconolactone isomerase	1.063829787	0.67568	0	1.63934	0.13255	2.083	0
Succinyl-CoA:3-ketoacid-coenzyme A transferase	2.127659574	0.67568	5.0847	39.3443	0.05681	0.453	0
Mandelateracemase/muconatelactonizase	3.191489362	10.1351	1.6949	0.81967	6.3435	10.33	5.6603774
Vanillin dehydrogenase	0	0	0	5.7377	1.60954	2.536	0
Vannilate transporter	2.127659574	0	0	4.09836	0.83318	4.167	5.0314465
Vanillate O-demethylase	0	8.10811	3.3898	6.55738	0.56807	8.062	5.0314465

Supplementary Figures (C)

Supplementary Figure S1. Phylogenetic affiliation of SSU rRNA sequences derived from human feces metagenomic DNA. Taxonomic distribution of SSU rRNA sequences affiliated with Bacteroidetes (a) and Firmicutes (b).

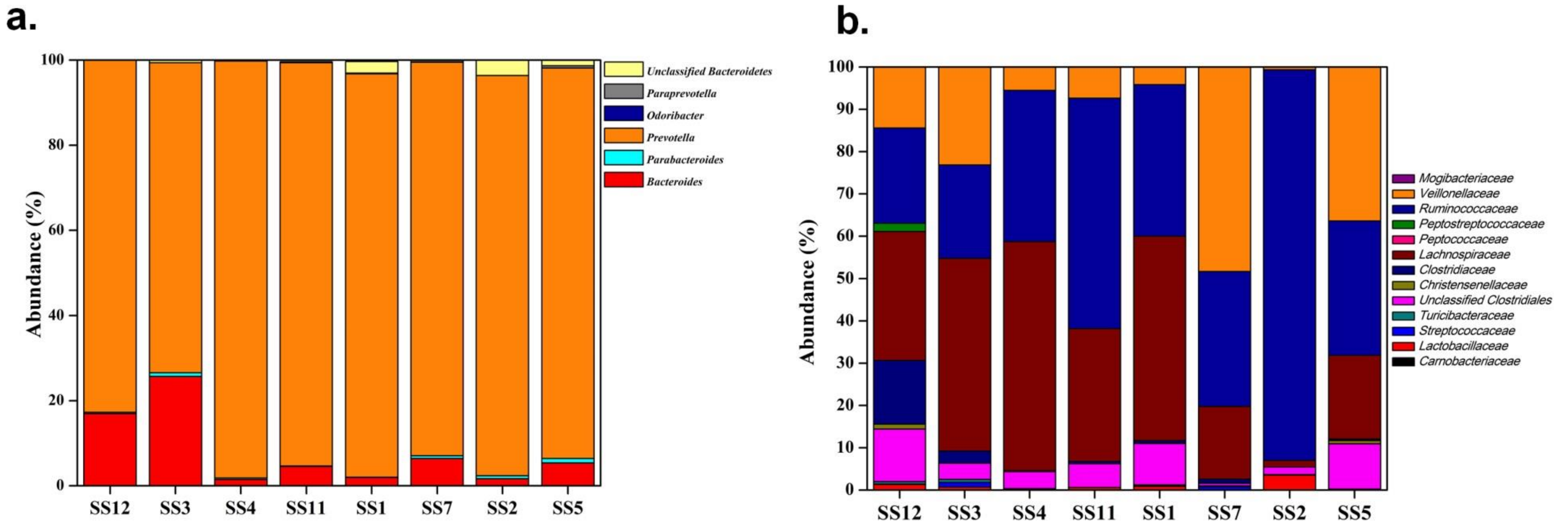

Supplementary Figure S2. Principle component analysis plots at different taxonomic levels (domain (a), phylum (b) and class(c) taxonomic levels).

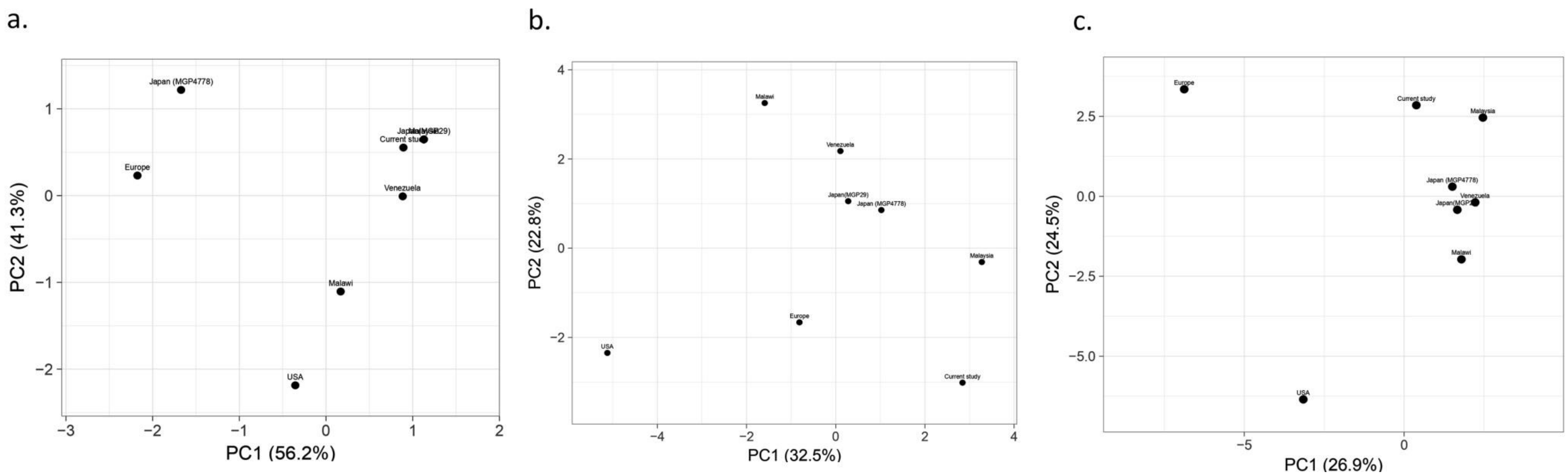

Supplementary Figure S3. Heatmap showing correlation of representative ribosomal features among metagenomic datasets at different taxonomic levels (domain (a), phylum (b) and class(c) taxonomic levels).

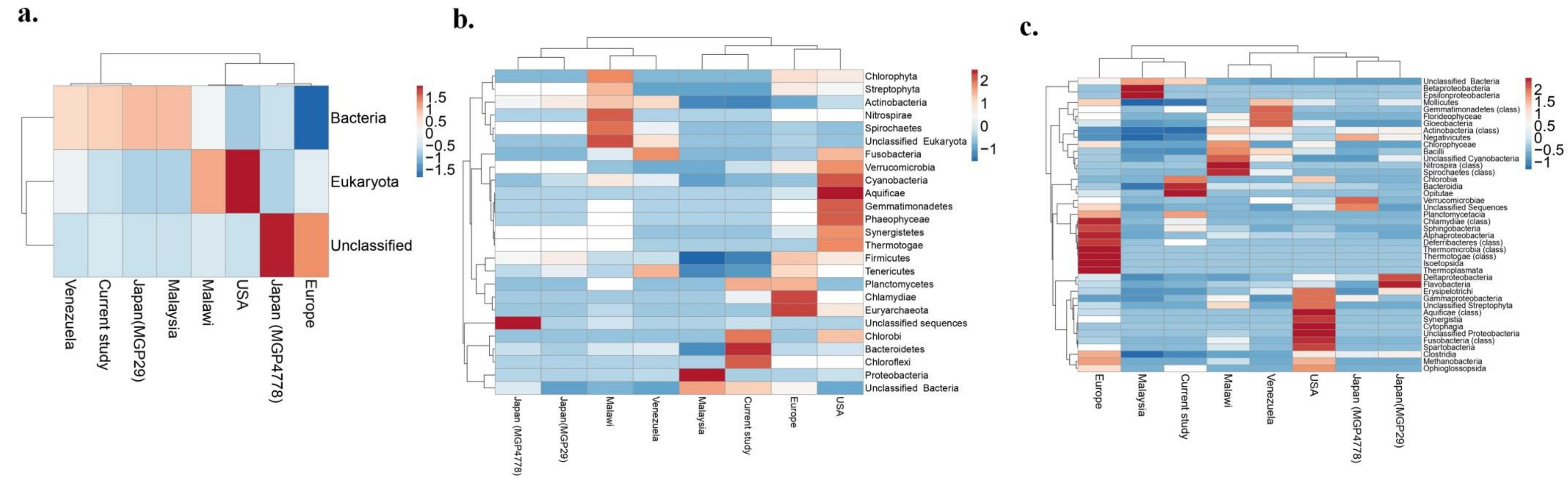

Supplementary Figure S4. The functional assessment of vanillin catabolism using LC-MS analysis from the microbial pellet incubated with 5mM vanillin at 37°C for 24hrs.

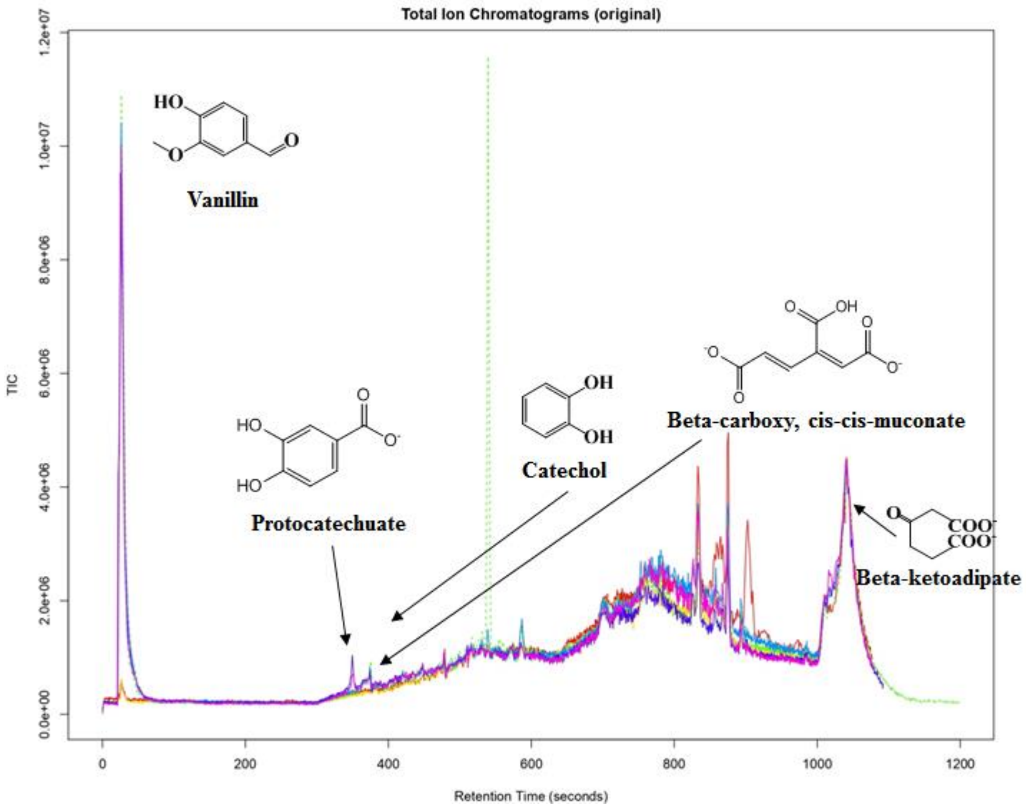

Supplement: Supplementary Figure 1 — Phylogenetic affiliation of SSU rRNA sequences derived from human feces metagenomic DNA. Taxonomic of SSU rRNA sequences affiliated with Bacteroidetes (A) and Firmicutes (B). [file Data_Sheet_1.pdf]
